# The behavioural variant frontotemporal dementia phenocopy syndrome is a distinct entity - evidence from a longitudinal study

**DOI:** 10.1186/s12883-018-1060-1

**Published:** 2018-04-28

**Authors:** E. Devenney, T. Swinn, E. Mioshi, M. Hornberger, K. E. Dawson, S. Mead, J. B. Rowe, J. R. Hodges

**Affiliations:** 1 Brain and Mind Centre, University of Sydney, Sydney, NSW 2050 Australia; 2 ARC Centre of Excellence in Cognition and its Disorders, Sydney, Australia; 3 Medical Research Council Cognition and Brain Sciences Unit, Cambridge, UK; 4 Faculty of Medicine and Health Sciences, University of East Anglia, Norwich, UK; 5 Department of Clinical Neurosciences, University of Cambridge, Cambridge, UK; 6 MRC Prion Unit, Department of Neurodegenerative Disease, UCL Institute of Neurology, Queen Square, London, UK

**Keywords:** Frontotemporal dementia, Phenocopy syndrome, Prognosis, Genetics, Cognition, Behaviour

## Abstract

**Background:**

This study aimed to i) examine the frequency of *C9orf72* expansions in a cohort of patients with the behavioural variant frontotemporal dementia (bvFTD) phenocopy syndrome, ii) observe outcomes in a group of phenocopy syndrome with very long term follow-up and iii) compare progression in a cohort of patients with the phenocopy syndrome to a cohort of patients with probable bvFTD.

**Methods:**

Blood was obtained from 16 phenocopy cases. All met criteria for possible bvFTD and were labeled as phenocopy cases if they showed no functional decline, normal cognitive performance on the Addenbrooke’s Cognitive Examination-Revised (ACE-R) and a lack of atrophy on brain imaging, over at least 3 years of follow-up. In addition, we obtained very long term follow-up data in 6 cases. A mixed model analysis approach determined the pattern of change in cognition and behaviour over time in phenocopy cases compared to 27 probable bvFTD cases.

**Results:**

All 16 patients were screened for the *C9orf72* expansion that was present in only one (6.25%). Of the 6 cases available for very long-term follow-up (13 - 21 years) none showed progression to frank dementia. Moreover, there was a decrease in the caregiver ratings of behavioural symptoms over time. Phenocopy cases showed significantly slower rates of progression compared to probable bvFTD patients (*p* < 0.006).

**Conclusion:**

The vast majority of patients with the bvFTD phenocopy syndrome remain stable over many years. An occasional patient can harbor the *C9orf72* expansion. The aetiology of the remaining cases remains unknown but it appears very unlikely to reflect a neurodegenerative syndrome due to lack of clinical progression or atrophy on imaging.

## Background

The classical features of behavioural variant Frontotemporal Dementia (bvFTD) syndrome are well established. The current consensus criteria incorporates cognitive, behavioural, neuroimaging, genetic and pathological parameters, to provide a framework to make accurate diagnoses by ranking the level of diagnostic certainty as possible, probable and definite [1]. Although the accuracy of these criteria has been pathologically validated, controversy still exists regarding the aetiology, progression and prognosis of possible bvFTD [2]. A recent study which followed FTD patients over a five year period found that a number of possible bvFTD patients remain in this category for many years and appear not to progress on cognitive and behavioural measures [3]. A number of these patients are classified as ‘phenocopy syndrome’ cases [4–7]. Patients harboring the *C9orf72* expansion may also satisfy criteria for possible, but not probable, bvFTD at first presentation and may be atypical with pervasive psychotic features [3]. Moreover, cases who have been labeled as the ‘phenocopy syndrome’ have also been reported to carry the *C9orf72* expansion [8, 9]. The question remains, just how many of the phenocopy cases have the expansion?

The present study sought to address this issue by exploring the outcomes in a large and unique cohort of phenocopy patients that have been followed over many years and screened for the *C9orf72* expansion. A mixed model analysis was employed to determine the rate of change in global cognition and behaviour over time in these phenocopy cases compared to a group of patients with probable bvFTD.

## Methods

### Patients

Patients were assessed at the specialist early-onset dementia clinic at Addenbrooke’s Hospital Cambridge between 1993 and 2007. Patients who satisfied criteria for possible bvFTD only, and were seen on at least two occasions; with initial and follow-up evaluation at least 3 years apart, and in whom blood had been obtained for gene screening, were included in the study. Patients were excluded from the study if they progressed to probable bvFTD over the study period. Exclusion criteria also included a current or past medical history of a psychiatric condition, traumatic brain injury, drug or alcohol abuse and cerebrovascular disease. Of note patients who experienced delusions or hallucinations were included in the study.

Of the 16 cases, three were still under regular review in the clinic in 2014. We attempted to contact the remainder and we able to reassess three additional cases. Thus very long term follow-up (ranging from 13 to 21 years) was available in 6 cases.

A group of probable bvFTD patients were included in the study to serve as a comparison group for the mixed model analysis, to determine differences in progression rates. These patients (*n* = 27) were assessed at FRONTIER, a frontotemporal dementia specialist research clinic and met probable diagnostic criteria for bvFTD. They were matched for age, sex and education to the phenocopy cases. Patients with probable bvFTD who were subsequently found to carry the *C9orf72* expansion were not included in this group. None of these patients carried a *GRN* or *MAPT* mutation. The results below relate to the phenocopy cases only unless otherwise stated.

Patients were classified according to the current international diagnostic criteria [1]. Patients were classified as possible bvFTD when they met three of the six core behavioural features of bvFTD, but had normal brain imaging and an absence of typical genetic or pathological findings. Probable bvFTD, criteria was met when patients firstly satisfied possible criteria with additional evidence of functional decline, and frontal or temporal abnormalities on MRI or Fludeoxyglucose (18F)-Positron emission tomography (FDG-PET) [1]. In this study MRI scans were performed in all cases and a validated visual rating scale, assessed atrophy of the orbitofrontal cortex, anterior temporal poles and insular cortex, according to previously published data [10, 11]. Atrophy was rated on a Likert scale by a blinded rater after appropriate training on an independent data set. Intra-class correlation coefficient to assess inter-rater reliability was very high (Cronbach’s alpha = .9).

### Clinical assessment

A comprehensive clinical assessment was conducted with the patient and behavioural symptoms were explored with the carer using the CBI (Cambridge Behavioural Inventory), [12]; a higher score indicates greater impairment (maximum score – 316). Global cognitive function was measured using the Addenbrooke’s Cognitive Examination-Revised (ACE-R) [13]; a normal score > 88/100.

### Genetic screening

Blood samples were screened at the Medical Research Council Prion Unit, London, or at NeuRA, Sydney, for the *C9orf72* expansion based on the repeat-primed polymerase chain reaction technique as previously described by Renton [14]. Genomic DNA was extracted from blood according to standard procedures. Samples were scored as expansion-positive if they harbored > 30 repeats. *C9orf72* hexanucleotide repeat non-expansion alleles were detected by polymerase chain reaction amplification and capillary electrophoresis.

### Statistical analysis

Data were analyzed using SPSS 22.0 statistical package. Normal distribution was determined by means of Kolmogorov-Smirnoff tests. Parametric variables were compared across groups via independent t-tests and analysis of variance (ANOVA). Non-parametric data were analyzed using Mann-Whitney and Kruskal-Wallis tests, and Chi-Square tests compared categorical data. Linear mixed effect models examined change in performance over time [15]. Such measures are useful in these circumstances as they take into account the variability in follow-up time within the phenocopy and probable bvFTD groups, and the significant difference in follow-up between the two groups.

## Results

### Patients

**Fig. 1:**
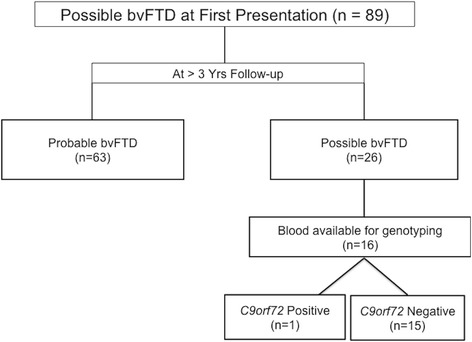
Longitudinal changes in diagnosis and genetic findings. Flowchart demonstrating the number of patients from the Cambridge cohort at presentation with possible bvFTD, according to diagnostic criteria for bvFTD, and the change in diagnosis and subsequent genetic findings over the follow-up period

Between 1993 and 2007 a total of 89 patients with possible bvFTD were assessed and followed for at least 3 years in the specialist clinic. Of these 89, a diagnosis of probable bvFTD became apparent on follow up in 63 (Fig. 1). The remaining 26 were given a label of phenocopy syndrome on the basis of a lack of progression with relative preservation of activities of daily living, maintained performance on the ACE-R and a normal MRI as assessed by a validated visual rating scale.

Within the comparison bvFTD group, four patients have now come to autopsy and each of these patients showed FTLD pathology including TDP-43 in one, TAU in another and FUS in another.

Blood sampling was obtained in all cases attending the clinic in 2007. In the phenocopy cohort of 26 cases, blood was available in 16 for genotyping.

### Genetic testing

The *C9orf72* expansion was present in one of 16 patients who had blood obtained for genotyping, representing 6.25% of the cohort. This patient was male and in his 60’s when he presented with a two-year history of behavioural change. At presentation his score on the CBI of 152 was very high and on the ACE-R his score of 81 was just below the cut-off of 88. A MRI scan was normal. When last seen in 2006 scores, had improved with a CBI score of 136 and an ACE-R score of 89. An FDG-PET scan showed no areas of significant brain hypometabolism. He was then lost to follow up in 2008 (11 years after onset) and died of an unrelated condition in 2010. Unfortunately post mortem brain examination was not performed.

### Cognitive and behavioural measures at baseline and follow-up

The 16 phenocopy patients with available genotyping comprised 15 men with a mean age of 55.7 (range 47 to 69 years). Twelve of the 16 were under 65. The mean follow-up time was 8 years. Table 1 demonstrates the baseline demographic information for these 16 cases and the comparison group of probable bvFTD patients and includes the mean ACE-R and the CBI scores on first assessment and length of follow-up for the phenocopy and the probable bvFTD group.

**Table 1:** Phenocopy cases – demographic details

Demographics at Presentation	Phenocopy bvFTD (*n* = 16)	Probable bvFTD (*n* = 27)	*P* value
Age at Onset, yrs	55.7 ± 6.3	59.7 ± 8.1	0.1
Sex (M:F)	15:1	22:5	0.4
Disease Duration, yrs.	3.9 ± 2.3	3.8 ± 2.4	0.1
Education, yrs.	11.4 ± 2.1	12.4 ± 3.4	0.3
ACE-R	89.2 ± 6.4	72.8 ± 14.6	0.001
CBI	91.3 ± 59	72.5 ± 21.5	0.3
Follow-up, yrs.	7.3 ± 4.2	3.2 ± 1.3	0.002

Demographic information (Mean ± standard deviation scores) for the phenocopy cases with blood available for *C9orf72* expansion testing, and probable bvFTD cases. *bvFTD* behavioural variant frontotemporal dementia, *ACE-R* Addenbrooke’s Cognitive Examination-Revised, *CBI* Cambridge Behavioural Inventory

At presentation 10 of the 16 phenocopy patients scored above 88 on the ACE-R, and none of the remainder scored below 80/100. At last follow-up six of these patients still scored within the normal range. The profile of behavioural symptoms at presentation and last follow-up was typical of bvFTD with high endorsements for motivation (apathy), stereotyped and abnormal behaviours, changed appetite and eating and mood.

At presentation there was a significant difference in the ACE-R scores between the phenocopy cases and the probable bvFTD group (*p* = 0.001); the mean ACE-R score in the phenocopy group was 89/100, whereas the mean score for the probable bvFTD group was 73/100. In contrast both groups had equivalently high scores on the CBI (*p* = 0.3).

**Fig. 2:**
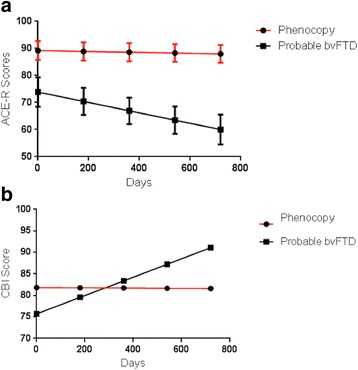
Longitudinal changes in ACE-R and CBI – phenocopy and bvFTD cases. **a** demonstrates estimated marginal means based on the % change in ACE-R score across time for phenocopy and probable bvFTD cases. Time (*p* < 0.001). Time x Diagnosis (*p* = 0.006). **b** demonstrates estimated marginal means based on the change in CBI scores across time. Time (*p* < 0.001). Time x Diagnosis (*p* = 0.06)

The phenocopy group was then compared to a group of probable bvFTD cases using a mixed model analysis that took into account the variability of follow-up within and between the two groups. On a measure of global cognitive function, the ACE-R, the groups combined showed significant deterioration over time (*p* < 0.001) with a significant interaction between disease group and time (*p* = 0.006) indicating a faster rate of decline in probable bvFTD cases compared to phenocopy cases (Fig. 2). On a measure of behaviour, the CBI, the group as a whole showed significant deterioration over time (*p* < 0.001), however while the interaction between disease group and time was not significant there was a statistical trend (*p* < 0.06) suggesting a faster deterioration in behaviour in the probable bvFTD group compared to the phenocopy group.

The mean ACE-R and CBI scores with 95% confidence intervals, calculated according to the mixed model statistic, for standard times intervals, are demonstrated for the ACE-R and CBI in Table 2. Table 2 also shows the mean ACE-R and CBI scores at last follow-up for the phenocopy and probable bvFTD group; these are for illustrative purposes only as the follow-up times were variable within and between the groups and therefore statistical analysis based on these measures is not appropriate.

**Table 2:** Phenocopy and bvFTD cases - Longitudinal changes in ACE-R and CBI

Time	Phenocopy bvFTD (*n* = 16)	Probable bvFTD (*n* = 27)
ACE-R – Scores (mean, 95% CI)		
Day 1	92 (84.1-99.9)	72 (65.7-78.3)
Day 180	91.6 (84.1-99.2)	69.5 (63.8-75.2)
Day 360	91.3 (84-98.6)	67 (61.5-72.5)
Day 540	91 (83.8-98.1)	64.5 (58.8-70.2)
Day 720	90.6 (83.7-97.5)	62 (55.7-68.2)
CBI Scores (mean, 95% CI)		
Day 1	81.8 (62.7-101)	75.7 (60.7-90.7)
Day 180	81.8 (63.4-100.1)	79.6 (66.1-93)
Day 360	81.7 (64.1-99.4)	83.4 (70.5-96.3)
Day 540	81.7 (64.6-98.8)	87.2 (73.9-100.6)
Day 720	81.6 (65-98.2)	91.1 (76.3-105.9)
Last Follow-up (mean, SD)		
ACE-R	85.1 ± 7.1	54.2 ± 23.6
CBI	77 ± 47.3	93.9 ± 33.7

Follow-up data according to the mixed model analysis with standard time intervals generated by the model, for phenocopy and probable bvFTD cases. Bottom rows show the mean ACE-R and CBI scores at last follow-up. *CI* confidence interval, *SD* standard deviation, *bvFTD* behavioural variant frontotemporal dementia, *ACE-R* addenbrooke’s cognitive examination-Revised, *CBI* Cambridge behavioural inventory

### MRI at baseline and follow-up

Grey matter density was judged as normal (0) or within normal range (1) in the orbitofrontal cortex, anterior temporal poles and insular cortex in each of the phenocopy cases at baseline and at follow-up.

### Long-term clinical follow-up

In 2013 we attempted to contact the caregivers of the 15 living cases by post and to arrange a telephone interview. Three were still attending the clinic at regular intervals and we were successful in another three (total six), with lengths of follow-up ranging from 13 to 21 years from first visit to the clinic. All were living at home; 3 remained in the same relationship as at their presentation, and there had been no cases with progression to frank dementia.

## Discussion

This study provides evidence for the validity of the bvFTD phenocopy syndrome. Only one of 16 phenocopy cases (6.25%) had the *C9orf72* expansion and it is interesting to note that this is the only patient in the cohort who is known to have died. This study had the benefit of very long-term follow-up information, between 13 and 21 years, in 6 cases. There was no evidence of progression to frank dementia in any of the phenocopy cases over many years of follow-up.

The underlying aetiology of the phenocopy syndrome is unknown. On a clinical level, these patients present with cognitive and behavioural changes, that are identical to the deficits seen in probable bvFTD cases, yet do not show significant brain atrophy [6, 16]. Furthermore, a previous clinicopathological study found that 2 phenocopy cases did not have FTLD pathology at autopsy [17]. While it is possible that the phenocopy syndrome represents a late onset decompensated developmental disorder in the Asperger-Autism spectrum, it remains to be proven. In keeping with this hypothesis, such patients, although scoring normally on tests such as the ACE-R and measures of memory, may show mild deficits on tests of inhibitory control and emotion processing [18] as do patients on the Asperger-Autism spectrum [19, 20]. A recent study comparing phenocopy and probable bvFTD cases showed a high rate of adverse life events, relationship problems and cluster C personality traits comprising the avoidant, dependent, and obsessive-compulsive personality traits [21]. Putting these findings together it seems highly likely that the phenocopy syndrome is a final common pathway for a complex interaction of a number of personality and psychiatric factors.

Interestingly long-term assessments show that, although some patients continue to exhibit behavioural symptoms, these symptoms are rated as less marked by caregivers over time. This could, of course, simply reflect the fact that family members adjust to and are less troubled by the symptoms. A study of possible bvFTD patients followed over several years showed that a subgroup, many of whom had the *C9orf72* expansion, progressed on cognitive and functional measures while others, who lacked the expansion, demonstrated no change and conformed, therefore, to the phenocopy syndrome [3]. Although the former work did not have the benefit of such long-term follow-up, it mirrors the results from this study, which showed that the phenocopy cases did not progress on the CBI, and together these findings point towards a non-progressive non-neurodegenerative aetiology in phenocopy cases. The probable bvFTD patients were also significantly more impaired on the ACE-R at presentation, and the mixed model analysis revealed a significant deterioration in ACE-R scores over time in the probable bvFTD group compared to the phenocopy group further demonstrating the relative cognitive stability of phenocopy cases.

Studies of FTD have established that the *C9orf72* expansion, whilst variable in prevalence around the world, is a common Mendelian genetic cause of familial disease, and is also present in a proportion of sporadic cases [22]. The full clinical spectrum associated with the expansion is not yet clear but it has been shown that such patients have a high rate of psychotic symptoms and that there is considerable variability in the rate of progression. While some patients present with a long insidious history of gradual decline others have a more fulminating illness [8, 9]. Studies have also linked *C9orf72* to other clinical phenotypes outside of FTD and MND, including Parkinson’s disease, multiple system atrophy (MSA) and Alzheimer’s disease (AD), although many lacked neuropathological confirmation [22]. Nonetheless, there does appear to be partial penetrance as *C9orf72* carriers may remain asymptomatic into their 80’s [23]. We have confirmed that patients with the phenocopy syndrome may also harbor the expansion but in a well-characterized cohort with long term follow up this appears to be the exception. Interestingly, the only *C9orf72* carrier in our phenocopy cohort did not show any abnormalities on MRI or FDG-PET, in keeping with reports from prior studies [8, 9]. Our work provides data to support the informed genetic counseling of this clinical group. A lack of understanding of the phenocopy syndrome and support for the patients and their families can make recruitment into a research programme difficult. Nonetheless further work is necessary to confirm the proportion of the FTD phenocopy syndrome that has a genetic aetiology and also to confirm the underlying pathology in these cases. Moreover, study of the phenocopy syndrome may help clarify the link between psychiatric illness and frontotemporal dementia. As in this project, cases that have a psychiatric history are usually excluded from studies however this design may need to be reconsidered in the future in light of this apparent link and co-existence of psychiatric and neurodegenerative disorders.

## Conclusion

We propose that the phenocopy syndrome is a valid entity. These patients are almost always male and experience symptom onset between the ages of 45 and 65. Despite reported behavioural changes, they perform relatively normally on general cognitive tests such as the ACE-R or ACE-III, have preserved basic activities of daily living, lack atrophy on MRI and critically show no decline after 3 years of follow-up. Within the first two to 3 years of evaluation of possible bvFTD cases physicians should exhibit caution in diagnosing the phenocopy syndrome, since the majority will progress to probable disease over time and almost one half will progress within the first 3 years. It should be also stressed that although phenocopy cases may not harbour underlying neurodegenerative pathology, this is not a benign condition and caregiver burden can be high.

## Abbreviations

ACE-R: Addenbrookes cognitive examination – Revised
bvFTD: behavioural variant frontotemporal dementia
CBI: Cambridge behavioural inventory
FDG-PET: Fludeoxyglucose (18F)-Positron emission tomography
